# Aura Mapping: Where Vision and Somatosensation Meet

**DOI:** 10.3390/vision5040052

**Published:** 2021-10-30

**Authors:** Frances Wilkinson

**Affiliations:** Centre for Vision Research & Department of Psychology, York University, Toronto, ON M3J 1P3, Canada; franw@yorku.ca

**Keywords:** migraine, visual aura, somatosensory aura, cortical spreading depression, observational study

## Abstract

While migraine auras are most frequently visual, somatosensory auras are also relatively common. Both are characterized by the spread of activation across a cortical region containing a spatial mapping of the sensory (retinal or skin) surface. When both aura types occur within a single migraine episode, they may offer an insight into the neural mechanism which underlies them. Could they both be initiated by a single neural event, or do the timing and laterality relationships between them demand multiple triggers? The observations reported here were carried out 25 years ago by a group of six individuals with migraine with aura. They timed, described and mapped their visual and somatosensory auras as they were in progress. Twenty-nine episode reports are summarized here. The temporal relationship between the onset of the two auras was quite variable within and across participants. Various forms of the cortical spreading depression hypothesis of migraine aura are evaluated in terms of whether they can account for the timing, pattern of symptom spread and laterality of the recorded auras.

## 1. Introduction

Migraine aura has long attracted attention and investigation, both in the search for clues to the pathophysiology of migraine headaches [1,2] and as an example of endogenously driven conscious experience [3,4]. Visual auras are the most common form [5] and generally the most dramatic. As summarized recently by Schott [6], a rich source of information about the visual aura experience comes from meticulously produced drawings made during auras, a literature dating from the early recordings by Airy and others [7] to a series of 1000 aura drawings analysed quite recently [8]. Lashley [9] was perhaps the first to recognize the value of the carefully documented visual aura sequence in providing an understanding of cortical visual system organization, and Leão [10] was the first to note the similarity between the spread of visual and somatosensory aura symptoms and the slow movement of the wavefront of spreading depression through cortical tissue. Grafstein [11] documented the brief burst of intense neural activity preceding the neural depression at the wavefront, which might explain the positive aura features. Cortical spreading depression (CSD) is now widely accepted as the neural underpinning of the visual aura, at least those auras with the well-known fortification properties suggestive of orientation-tuned neurons in the primary visual cortex. The now-classic study by Hadjikhani et al. [12] provided vivid support for this hypothesis and heralded what was expected to be a new era of migraine aura investigation using various forms of brain imaging. Indeed, numerous studies have imaged the brains of migraineurs (reviewed by [13,14]), although few have had the capability and perseverance necessary to capture auras in progress.

It has long been appreciated that, while most common, visual aura is not the only aura modality, and that among visual auras, the classic fortification pattern described by Lashley and others is far from the only aura experience [5,15,16]. Somatosensory symptoms have been reported in many studies [5,16,17], and are most commonly experienced as spread across parts of the body surface of tingling (“pins and needles”), numbness or a combination of the two. This is not at all surprising since, as has been well appreciated since the time of Penfield [18,19], the body surface is represented topographically in the somatosensory cortex along the posterior bank of the central sulcus and on the post-central gyrus. The body areas most frequently reported to be affected are those with the largest cortical representation, the fingers and hands and the face, especially the mouth area [16]. The legs and trunk are more rarely mentioned, probably because much less cortical tissue is devoted to their representation, and possibly also because of their location in the maps (on the crown and the medial surface of the hemisphere). Auditory, olfactory and gustatory symptoms are seemingly quite rare, although all have been documented [20,21,22]. There are also numerous reports in the literature of more complex cognitive symptoms that often occur at some point during a migraine episode [23]. Most frequently mentioned are a range of language difficulties [24], which may include receptive language such as reading and comprehension of spoken language, and also language production problems including dysarthria (difficulty with articulation) and word-finding difficulties. More diffuse cognitive difficulties with concentration, and in some cases with memory, are also reported. There has been considerable recent interest in better describing this wide array of symptoms in the hope of better understanding how these phenomena are related to the more fully documented sensory auras [16,22,25].

It is also well known that many people experience more than a single manifestation of migraine aura. An individual may report both visual aura and somatosensory symptoms, and confusion or word-finding difficulties before their visual aura starts, as it progresses or after it ends [16,17,22]. Therefore, an obvious question is, and has long been, does this reflect a single spreading process moving through cortical tissue or does it represent a series of similar “outbreaks” of CSD or some similar process? And if a single one, where does it start? Hadjikhani and colleagues [12] described a wave of spreading depression beginning in area V3a and gradually engulfing all primary visual areas. A number of recent prospective studies have begun to address the issue of successive auras directly [16,17,23].

More than twenty-five years ago, the author’s laboratory pursued a similar question. Although most of our research on vision and migraine was psychophysical in nature and was carried out interictally in a laboratory setting (e.g., [26,27]), our earliest studies attempted to capture the properties of visual migraine auras as they progressed [28,29]. We were aware from Olesen’s group [30] that migraine retrospective reports on such seemingly simple issues as location of the aura in the visual field (left vs right) or side of headache are not reliable, and our own observations confirmed this conclusion. Therefore, over the course of several years in the 1990′s we recruited a population of individuals with migraine aura and trained them to document and map their visual auras as they were in progress. Initially, we did not gather specific details on other sensory effects beyond having participants note their occurrence. With time, we realized that several of our participants were experiencing somatosensory as well as visual symptoms during some episodes and it became clear that understanding the precise temporal relationships among these phenomena, along with the laterality of sensory effects might be a valuable route to identifying the source(s) of an aura episode. To this end, we began recruiting such individuals into a follow-up study, the principal goals of which were documenting the precise onset and offset times of the visual and somatosensory events and mapping the somatosensory events on maps of the body surface. Over the period 1993–1997, we collected multiple observations from six such individuals. By 1997, it was evident that brain imaging would soon offer a route to making more precise determinations of cortical distances within individual brains, something that could only be crudely estimated when this study began. The author had hoped to recruit additional participants, and collect further episode reports and possibly imaging data. However, due to events that frequently occur in academic careers (funding fluctuation, move to different institution and city, shifting research priorities) this did not happen. In light of recent interest in the literature in multiple and complex auras [16,23,24,31], this data set does continue to have relevance. As all original records including informed consent and ethics approvals had been retained by the author, this special issue devoted to migraine aura seemed an appropriate opportunity to place these findings into the literature. 

## 2. Materials and Methods

### 2.1. Participants

The six participants in this study were recruited as part of our broader study on mapping migraine visual auras (VA study), which was initiated by the author when she was a faculty member at McGill University in Montréal Canada. All procedures described here received ethics approval from the McGill University Research Ethics Board, and conformed to the Declaration of Helsinki. Recruitment was largely by referral from local area neurologists, family practice physicians, and in some cases by word of mouth, with subsequent written confirmation of migraine diagnosis from the participant’s supervising physician, obtained with the written permission of the participant. Five of the six whose data are presented here were referred from a tertiary care clinic specialized in headaches; not surprisingly, these cases were complex and all had experienced multiple pharmaceutical treatment options. However, none were on prophylactic medication for migraine at the time of their participation, and none had neurological comorbidities with the exception of fibromyalgia in two cases. The participants described here first provided several episode descriptions of their visual auras before being transferred to the visual-somatosensory (V-SS) study on the basis of their aura descriptions on initial interview and notations about somatosensory symptoms (numbness or tingling) made in their visual aura reports. The data presented here are from six individuals who agreed to participate in this second study and who submitted at least one report that included timing of both visual and somatosensory auras. Summary information about the participants is provided in Table 1. Most of the participants in our Montréal research, including four of the six described here, were French-speaking. All interviews were conducted in French if this was the primary language of the participant, and French or bilingual versions of all forms and other test materials were provided. Intake interviews were conducted first by telephone, followed by a home visit by a fluently bilingual member of the research group.

### 2.2. Visual Aura Mapping Methods

While the results of the VA study are not the focus of this report, it is important to describe how these data were collected, and what information they provided about each of the participants. Participants were provided with sets of aura forms and visual maps and were instructed in their use at the time of their home interview. The visual maps were 43 by 28 cm and had a 40 cm string emanating from the fixation cross at the centre of the map with a button at the far end (Supplementary Figure S1). The participant was trained to attach the map to a convenient vertical surface (wall, refrigerator door) at the onset of an aura and use the string (button to forehead) to maintain the viewing distance of 40 cm throughout the aura mapping, which allowed us to ascertain the precise angular location in the visual field of the drawn auras. They were instructed to write down the time the aura started and then, while fixating the cross, to draw the aura on the map at approximate five min intervals and to label each drawing with the time. They were also asked to complete the accompanying aura form during and immediately after the episode (Supplementary Figure S2) which asked about aura and headache details. It was repeatedly emphasized to the participants that it was critical that they complete the forms and maps ONLY if they could do it during an episode, that it was very important not to do it from memory at a later time, and that they should mail the forms back to us immediately (stamped addressed envelopes were provided for this purpose). This instruction was reinforced in follow-up phone calls to the participants made to thank them for mailed-in reports and to clear up any uncertainties about the study. We tried to communicate that it was not how many episodes they mapped that was important to us, and if they missed some, that was not a serious problem; our interest was in what they were able to record as the aura was in progress. 

### 2.3. Methods for Visual-Somatosensory (V-SS) Study 

When recruited into the second study (V-SS), participants were given a new form (Supplementary Figure S3) along with visual maps and somatosensory maps (Supplementary Figure S1). We emphasized the particular importance of completing the form, being sure to record the onset and termination times of each aura and left/right symptom locations accurately, and also the importance of the new somatosensory maps. They were asked to make three somatosensory map recordings at 5–10 min intervals and to record the time at which each was made. Since the visual mapping was quite taxing and as we had visual data from most of them already, we instructed them to map their visual auras if possible, but that this was the least critical component. The same instruction about recording episodes (times and maps) only as they were in progress was emphasized repeatedly. The new form also asked whether headache occurred, and whether it was bilateral or limited to the left or right side of the head. 

## 3. Results

Between March 1994 and February 1998, we received 50 episode reports from the six individuals (range 2–14); we also had 47 visual aura reports from the same individuals (range 2–22) from the earlier part of the project beginning as early as August 1993. Of the 50 reports for the V-SS study, 11 described episodes with only visual aura, and six episodes with only somatosensory aura. Of the remaining 33, 29 provided sufficiently complete data on the critical details of the episodes to be included in the V-SS results; the other four contained inconsistencies or omissions that prevented us from determining the relative timing of the auras. For brevity, and to avoid confusion, the terminology “P =” will be used to indicate the number of participants and “E =” to indicate the number of episodes in which a particular effect was seen. Individual participants are referred to by number as in Table 1 (e.g., P1).

In Supplementary Table S1, each of the 29 episodes is evaluated in terms of the current criteria for migraine with aura, as detailed in the 3rd Edition of the International Classification of Headache Disorders (ICHD-3) [32]. Since the ICHD-3 requires at least three of a list of six criteria to be met, we then evaluated each episode by this standard. Twenty-seven of the 29 episodes were found to meet the migraine with aura criteria (3 or more YES). In one episode (rated PROBABLE), the individual (P3) fell asleep 25 min into his second (visual) aura after taking sumatriptan and anti-nausea medication and awoke 2 h later with a severe headache and no visual aura symptoms, so it was not possible to determine with certainty either the length of the visual aura or the gap between aura and headache. Finally, a single episode (P6, Episode 5) was rated NO overall, as both auras were extremely long, consisted of negative symptoms only, and showed little evidence of local spread over the period mapped.

The criteria which were rated NO varied across participants, but some common patterns were seen. Firstly, somatosensory auras tended to be quite prolonged, 16 of the 29 exceeding 1 hr in length (P = 3) and lasting several hours in some instances. To the contrary, only 2 visual auras (P = 2) lasted longer than 1 h. The criterion of least one aura being unilateral failed in 7 episodes (P = 2). The criterion of at least one aura symptom being positive failed in 3 episodes (P = 1) in which some form of scotoma or obscured vision occurred with somatosensory numbness; this could not be evaluated in several episodes in which inadequate qualitative detail was provided. Finally, in three episodes (P = 1) the two auras began simultaneously rather than sequentially. In all three episodes, the somatosensory aura outlasted the visual aura.

### 3.1. Initiation Offset Time (IOT) and Laterality

In Table 2, each participant’s episodes among the dataset of 29 are listed in terms of two critical factors. The upper panel (Table 2a) is the time between the first detection of the two auras, which we will call initiation offset time (IOT), and its direction. Positive values indicated that the visual aura began before the somatosensory aura; negative values, the reverse. We emphasize that IOT refers only to initiation; in many cases, the first aura was still in progress when the second began. For four individuals, the onset of the visual aura always preceded the onset of the somatosensory symptoms. One individual reported only one episode, in which the somatosensory symptoms appeared first. For the final participant, the somatosensory aura preceded the visual aura (E = 6) or began simultaneously with visual symptoms (E = 3). The precedence of visual or somatosensory auras agreed with the retrospective descriptions provided during the intake interviews. While this order was quite consistent for each individual, the range of offsets recorded was large (0 to 75 min for the 28 auras scored as meeting ICHD-3 criteria), and each participant who submitted more than a single record showed a considerable spread of IOT values. The smallest range was 2–23 min (P1), and the largest, 2–75 min (P4). 

Table 2b lists the side (of the visual field, body, head) that the participant reported as the location of aura and headache symptoms. These are listed as either L (left), R (right), or B (bilateral). It is important to note that the sequence is always Visual-Somatosensory-Headache regardless of the order in which these occurred, which is addressed separately in Table 2a and Supplementary Table S1. This arrangement was used to facilitate easy comparison within and between individuals of auras of the same modality.

Only seven of the 29 episodes documented here consisted of two unilateral auras affecting the same hemifield and body side, and therefore likely reflecting activities within a single hemisphere of the brain. This would allow the possibility that a single wave of CSD might explain both auras, providing the timing can be accounted for, a topic we will take up in the Discussion. All remaining episode types require at least one additional mechanism to account for the phenomenology. 

**Figure 1:**
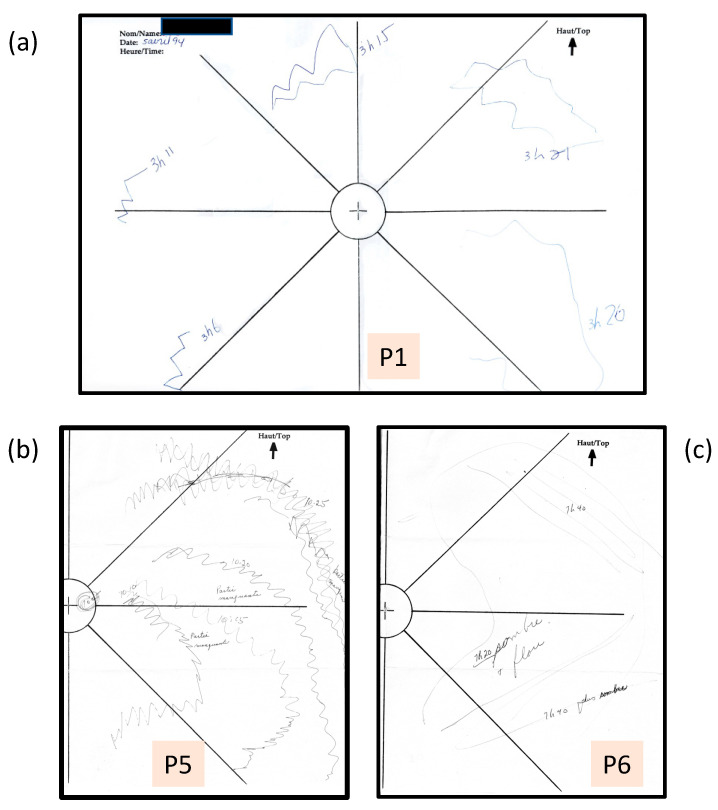
(**a**) P1: Example of a full aura map tracking a visual aura at five intervals between 3:06 and 3:26 pm. When in use, a 40 cm string attached to the centre of the central fixation cross (not shown here) was used to maintain the correct viewing distance. The observer drew the aura patterns while fixating the + at the centre of the map and labelled each observation with the time. (**b**). P5: One example of a fortification aura in the right visual field drawn between 10:05 and 10:25 am. Only half the map is shown as nothing was recorded in the left visual field. Missing parts (“partie manquante”) are indicated in the wake of the positive symptoms. (**c**) P6: An example of a negative aura drawn at two time intervals, noting that the drawn region is dark and blurry (“sombre and flou”), and at 7:40 darker (“plus sombre”).

When visual auras occurred first (4 participants), they were almost always unilateral (17/19 episodes), and the somatosensory symptoms that followed were also unilateral in most cases (15/19). Visual auras that followed (or began simultaneously with) somatosensory auras were split between unilateral (5/10; P6) and bilateral (5/10; P3 & P6) visual symptoms. The five bilateral visual auras were described or drawn as tunnel vision (peripheral vision loss/blurring/pixilating with central sparing); these always followed somatosensory auras involving both hemispheres. The five unilateral auras were similar in that they involved peripheral visual loss in the right hemifield (Figure 1c); all five followed somatosensory auras involving facial numbness on the right side (and mixed side effects in the hands).

### 3.2. Local Spread

#### 3.2.1. Visual

Positive and negative aura properties and average aura durations may be found in Table 3. The classic positive visual aura, often described in the literature as fortifications and by patients as zig-zags, shows clear spread across a visual hemifield, consistent with the topographic representation of the visual hemifield in the primary visual cortex [8,9,33]. Only one of our participants (P5) consistently reported visual auras of this pattern (Figure 1b). Her auras occurred in both left and right hemifields in different episodes, and typically showed clear spread from close to the fovea toward the periphery. The median duration of her visual auras was 25 min, which would be consistent with CSD spreading through V1 (primary visual cortex). A second individual (P1) reported a variant of the fortification aura consisting of bright zig-zags which moved around the visual field in an arc, and in some cases moved smoothly across the vertical meridian at the top of the field to form a circle around the entire visual field (Figure 1a). P2 also always reported positive auras: yellow spots or of flashing shards of light which moved about in the lower left visual quadrant but did not show systematic progress across the field. V1 contains a continuous mapping of the visual hemifield, whereas the secondary visual areas V2 and V3 are split along the horizontal meridian, such that the lower visual fields are represented on the upper lip of the calcarine fissure, superior to V1 and the upper visual fields on the lower lip, inferior to V1. Thus, the fortification auras described above, in which either the zig-zag wavefront is continuous across the horizontal meridian (P1) or the zigzags move smoothly from lower to upper field (P5) strongly suggest a V1 origin, while the auras mapped by P2, and limited to the inferior quadrant, could also arise from the superior portion of V2 or V3.

Two individuals (P4 & P6) reported purely negative auras in most episodes, consisting of dark clouds or grey areas moving across or obscuring parts of the visual field. P6 was able to provide crude maps of the spread of these grey zones. Her visual field loss affected the peripheral visual field(s), in some cases unilaterally as in Figure 1c; in other episodes, both hemifields were affected with the field loss usually beginning in the lower field(s) and spreading upward, in some instances leading to complete tunnel vision. Both P4 and P6 also reported at least one episode with positive symptoms as well as negative—either flashing, dazzling lights or, in one instance, a spreading fortification aura. Lastly, P3 described his auras as starting with bright flashing “rotating diamonds” in one hemifield, followed by gradual loss of usable vision spreading inward from the periphery bilaterally, giving rise to tunnel vision. He drew and described his peripheral vision as consisting of “ill-defined cells, that let light through but make information unresolvable”, “similar to a kaleidoscope without the interesting colours”. It should be noted that we have not made a distinction between negative symptoms and disturbances of visual perception (DVP [16]) because in our experience, it is very difficult to distinguish between true scotomas and dark, grey or blurred areas; these may as reasonably be considered weaker forms of depressed neural activity as some other form of disturbance. None of our participants described the more distinct distortions of vision (heat waves, line, shape or depth distortions, oscillopsia) included in the DVP category [16]. The one exception was P3 whose tunnel vision described above might fall into the category of fractured vision [16].

#### 3.2.2. Somatosensory 

Unlike the two-dimensional retina, the skin surface cannot be represented as a single continous sheet so the somatosensory cortex of necessity contains discontinuities in the map of the body. Penfield first elucidated this mapping with his famous homunculus [18,19] based on electrical stimulation in awake neurosurgical patients. The primary somatosensory cortex (SI) consists of four parallel cytoarchitecturally distinct strips (Brodmann areas (BA) 3a, 3b, 1, and 2) which together form the banks and the crown of the post-central gyrus. As one moves upward from the inferior tip of SI near the insula, the somatotopic map begins with the face (intra-oral structures, lips and then skin surface), followed by an abrupt transition to the hand (thumb, fingers, palm), and then arm (wrist to shoulder). Another transition follows from shoulder to the trunk, and finally to the lower limb with the toes occupying the extreme end of the SI strip on the medial surface of the brain. None of the auras reported here described continuous spread through the length of SI as might be predicted from a simple model of CSD. Instead, in every case they represented more localized areas of tingling or numbness, affecting the face, hand, arm or a combination of these regions. The trunk below the neck was never involved and the lower limbs, only twice. 

**Figure 2:**
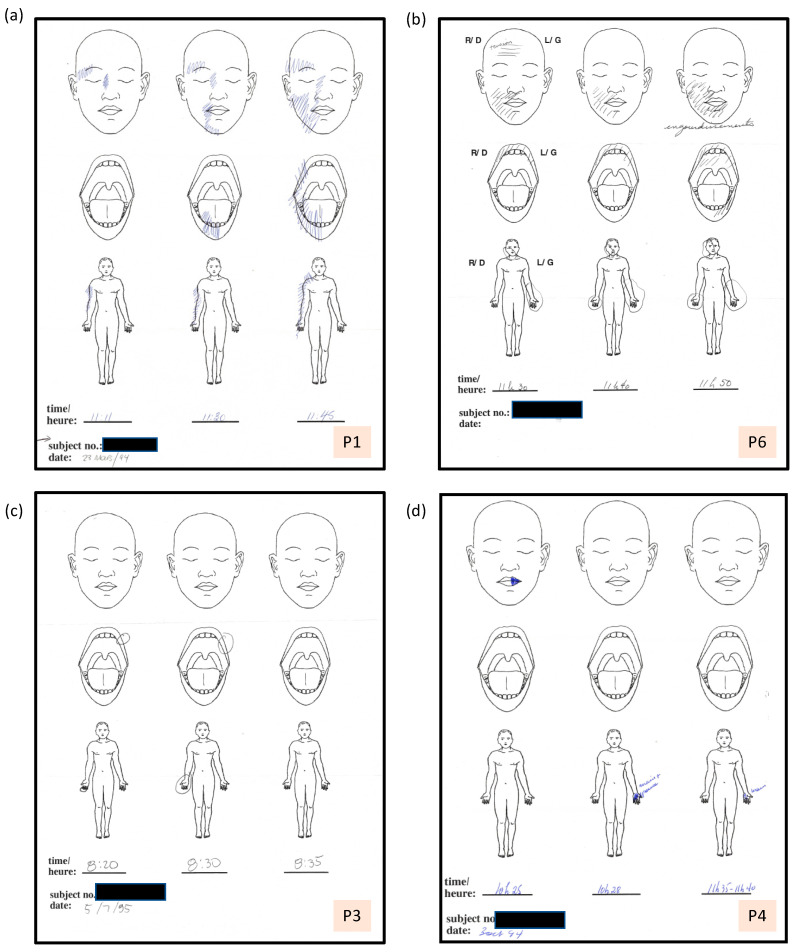
Sets of three somatosensory maps from single episodes recorded by four participants. (**a**) P1: Maps of unilateral aura symptoms on the face and arm (**b**) P6: Maps of a bilateral aura with mirror image regions of the hand affected beginning first on the left and more extensive on this side. The facial numbness (“engourdissements”) is largely on the right but spread across the midline to include the lower left lip and chin over 20 min. (**c**) P3: Somatosensory aura illustrating simultaneous aura symptoms in different body regions on opposite sides of the body. (**d**) P4: Unilateral aura showing localized symptoms in the lip, followed by the hand and thumb shortly after onset. The symptoms disappeared, and then reappeared briefly an hour later, as indicated in the third drawing.

P1 and P6 experienced prolonged somatosensory symptoms in both face and limb(s) and provided the most detailed records of the pattern of spread (Table 4 and Figure 2). P1′s facial symptoms were generally as illustrated in Figure 2a: beginning around the eye (inner nose, outer brow) and spreading down the outer edge of the nose encompassing the cheek and the lips and, in some cases, involving the tongue and teeth. They always respected the midline, restricted to one side of the face. At the same time she described symptoms in her arm, which could begin at the hand/wrist, elbow (Figure 2a), or shoulder and spread up and/ or down the arm, but did not appear to go beyond the shoulder into the trunk or lower limb. In episodes in which the symptoms began at the shoulder, the separation in SI from the site of the facial symptoms is estimated to be 3–4 cm.

P6 reported a similar pattern of facial symptoms (numbness) except that it generally began below the eye and more laterally in the cheek, moving toward the midline of the face and then into the lips as the aura progressed, in some cases spreading across the midline in the lips and mouth. Either simultaneously, or with a delay of a few minutes, she reported tingling in her hand(s), which generally began in the fingertips and spread to the hand and sometimes the forearm (Figure 2b). In 3/9 episodes, both her facial and hand symptoms were bilaterally symmetric. In the remaining cases, symptoms were purely unilateral (E = 2), or the limb symptoms began earlier and were more extensive in one limb than in the other (E = 4; Figure 2b). Slight spread across the midline of the face was also seen in these cases; however, the early/dominant side was not always the same in hand and face. Although her symptoms could last several hours, she never described them as moving beyond the forearm; however, she did note on several occasions that her hands were numb and she had difficultly picking up and holding onto objects, suggesting that in prolonged episodes proprioceptive or motor regions might also be affected. While the early symptoms in her fingers were always described as “tingling”, facial symptoms and long-lasting effects in the hands were labelled as “numbness”.

**Figure 3:**
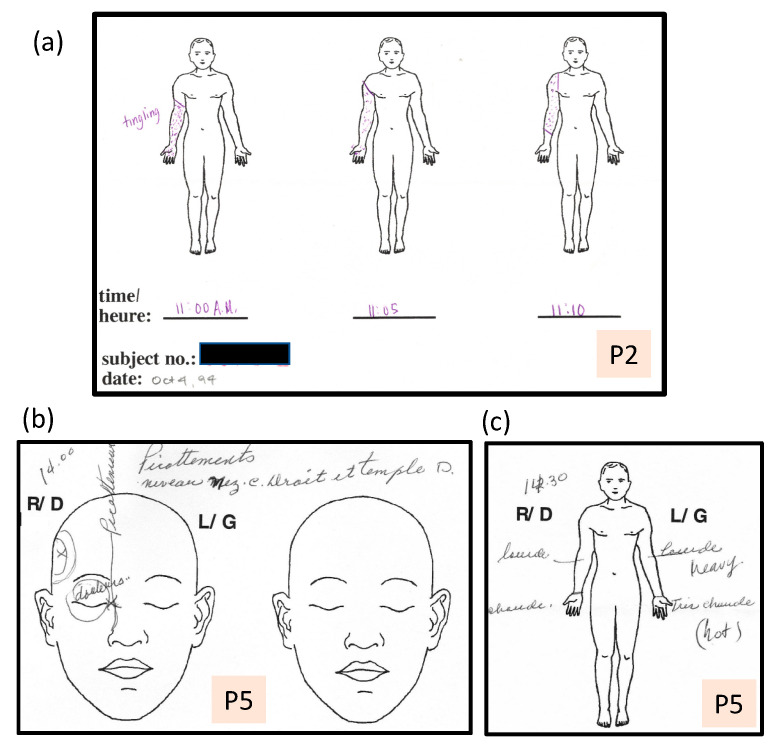
(**a**) P2: Unilateral aura affecting the arm and hand only, spreading to the shoulder over 10 min. (**b**) P5: An example of a somatosensory aura limited to tingling on the right side of her nose and at her right temple (“picottements niveau nez droit et temple d(roit)”), along with pain (“douleurs”) in the area of her right eye. (**c**) P5: 30 min later in the same episode, indicating heaviness (“lourd”) in both arms, and heat (“très chaud”) in both hands.

P3 and P4 reported much more limited effects in both hand and face. In both cases, symptoms were unilateral, affecting the lips on one side and either the contralateral (Figure 2c) or the ipsilateral (Figure 2d) hand. These symptoms began either simultaneously or within a very short period (3–5 min) and lasted no more than 15 min, dissipating by the final mapping. P4 also reported episodes involving hand symptoms only, and twice noted that while her hand was affected, the “usual wave” (spread of symptoms) did not occur. P2 (Figure 3a) experienced tingling that moved up the arm to the shoulder (but not beyond into the trunk) and began to resolve over the 10–30 min mapping; her face was never affected. As with P1, this pattern seems consistent with CSD moving through the region where the arm is mapped in SI. 

Lastly, P5 reported unique facial symptoms, tingling beginning on and generally limited to the bridge of her nose, usually on one side (Figure 3b). This symptom appeared to arise in close temporal contiguity with the onset of her headache, which was also frequently accompanied by neck pain and pain around the eye. In some instances, she described the tingling as moving up over the top of her head and down to her neck and/or to her temple but she did not provide enough temporal details to assess the rate of this spread. In three episodes she also described her arms (and in one case legs) as heavy and hot (Figure 3c), but again provided no temporal details. Her symptoms, although very localized, were reported to last well over an hour in most cases. 

In summary, aura symptoms in different body regions were not sequential; rather symptoms in the face and upper limb seemed to begin together or within a short time of one another. Locally, spread in the arm seems consistent with local CSD. Pattern of spread in the hand was not as well documented; this may reflect the complexity of the hand representation across the subregions of SI [34,35,36,37]. Most often the fingers were all affected together, suggesting spread across, rather than vertically in SI, as the thumb and individual fingers are represented separately moving upward from the face/hand border (at least in subregion BA3b) [34,35,36,37]. The sequence of facial symptoms in P1 and P6 (Figure 2a,b) suggests spread beginning in the border region and moving downward from this point into the face/oral region. In five episodes, two distinct trigger points are indicated, either because of spatial separation of affected regions within the cortical homunculus or because face and hand symptoms were limited to opposite sides of the body. In the remaining cases in which both facial and hand symptoms were reported, we cannot rule out a common trigger site near the border between the face and hand representations. 

### 3.3. Headache

Headache was reported for all 29 episodes. Only 16 reports provided headache onset times; an additional six reports noted the time, usually during the auras, when abortive medication was taken, suggesting either that a headache had begun or that one was expected soon based on previous experience.

Headache side showed no consistent relationship to either aura type. Bilateral headaches generally followed either bilateral auras or two unilateral auras occurring on opposite sides/visual fields; however, all possible combinations of unilateral headache and unilateral or bilateral visual and somatosensory aura may be found in Table 2b.

## 4. Discussion

**Figure 4:**
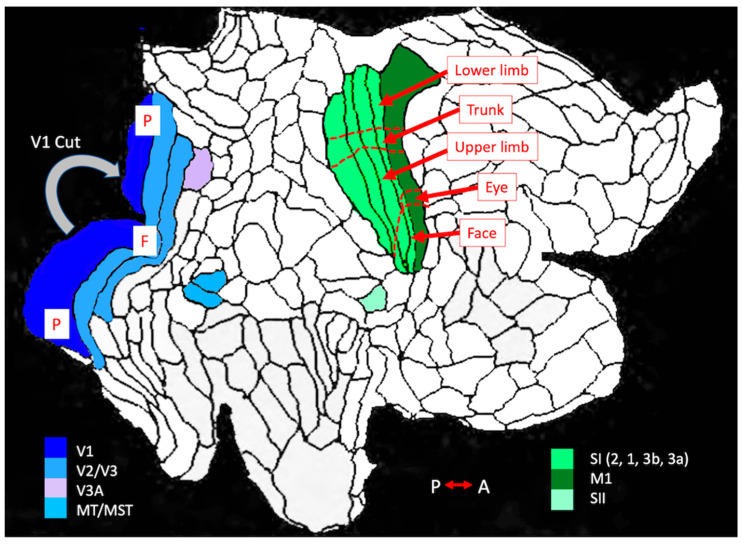
Flat map of human right hemisphere showing the parcellation of neocortex, adapted by permission from Springer Nature: A multi-modal parcellation of human cerebral cortex, Supplementary neuroanatomical results (Supplementary Figures 1 and 8), M.F.Glasser et al. Copyright 2016. Primary visual cortex (V1) in dark blue, with cut indicated. Secondary visual cortical areas V2 and V3 in light blue. The location of foveal (F) and peripheral (P) representations are indicated. Higher level occipital visual areas V3a (mauve) and MT/MST (turquoise). Primary somatosensory cortex SI, in medium green, consisting of BA3a, BA3b, BA1 and BA2 moving from anterior to posterior (A-P). Adjacent to BA3a is motor cortex (BA4) in dark green. The small light green area below and posterior to SI is SII, a secondary somatosensory area. The axis perpendicular to the A-P comprises shifts in both medial-lateral and superior-inferior directions in the three-dimensional brain. For simplicity in the text, the end of the somatosensory areas representing the hindlimb is designated as Top/Upper, and the face region as Bottom/Lower. Functional subdivisions of somatosensory and motor cortex are indicated, as described and illustrated by Glasser et al. ([38]; Supplementary neuroanatomical results Figure 8).

At the time that these reports were collected, our ability to assess distances through cortical tissue was severely limited, as it was based on published atlases which showed brain slices taken at specified spatial intervals. However, due to the enormous advances in imaging technologies in the intervening 25 years, we now have very detailed representations of the human neocortex. Functional imaging has also provided a whole new perspective on brain networks, no longer defined by close anatomical connection alone, but also by functionally defined correlations in activity. Most impressive in terms of mapping is the recently published work from the Human Connectome Project which has identified a total of 180 distinct cortical areas in each hemisphere, represented in flat-map presentations as well as on inflated brains ([38]; see Figure 4). This provides a much stronger basis for speculation about the localization of the effects described by our participants. Nevertheless, because we do not have structural or functional imaging data from these individuals, because there is reported to be quite a range in overall cortical area and in individual subregion areas [38,39] and because the nature of the flat-map process introduces inherent distortions [40], distances are still at best crude approximations. Moreover, Dahlem et al., in a recent modelling study [41,42], suggest that structural features of the highly convoluted human cortex may constrain the path of CSD, creating what they call labyrinths and hot spots that may be unique for each individual. With these caveats in mind, let us turn to the most important observations from our participants.

### 4.1. The CSD Hypothesis

#### 4.1.1. Aura Initiation Offset Times

The main question that drove this investigation was whether the temporal and laterality relationships between visual and somatosensory auras was consistent with the hypothesis of a single wave of CSD sweeping through the posterior cortex. Very crude estimates based on flat maps (Figure 4) would suggest that the approximate range of CSD wave travel times between the V1 (BA17) and anterior SI (BA3a&b) would be 40–60 min, and that at the shortest distance between somatosensory and visual cortex (posterior edge of SI (BA2) to the anterior edge of secondary visual cortex (V3), a CSD wave would require a minimum of 25–40 min. These times set the limits if one wished to test the hypothesis that a migraine aura initiated in one of these sensory area triggers a wave of CSD both through that region and spreading out across intervening cortex until it reaches the cortical representation of the second modality. This ignores the details of the experienced phenomenology, which in some cases might indicate considerably longer trajectories (e.g., the representation of the mouth to the representation of the lower peripheral visual field).

As described in Results (see Table 2b), a wide range of times was reported between initiation of the first and second auras, regardless which one occurred first. A similar range of timing between auras was reported in a recent prospective study [17]. Twelve of our 29 episodes were separated in onset by 25–75 min which might be consistent with spread from one primary sensory area to the other. Sixteen episodes had offsets of under 25 min, eight of which were 5 min or less. Equally important is the observation that similar ranges were seen for individual participants, and this was true for purely unilateral auras affecting the same visual field and body side, and for the within-hemisphere components of bilateral auras (e.g., left visual field, left hand). The short IOTs (<25 min) could potentially be explained by a single triggering event occurring at some point in the parieto-occipital cortex between somatosensory and visual cortex and spreading toward both. In this case, the IOT tells us little about the trigger location, only about how much closer it is to one sensory area than to the other. Very small offsets would suggest a trigger point roughly equidistant from the two areas, whereas a positive value of 15 min would indicate a trigger point considerably closer to visual cortex and a negative value, one closer to somatosensory cortex. Note that, in view of the possibility of variation in the path of spread in highly folded cortical tissue [36], “equidistant” actually means in time, not necessarily along a straight line in space. It has often been suggested that CSD spreads “silently” through cortical regions that are not primarily sensory—perhaps even in migraine without aura [43]—and such spread has been offered as an explanation of some of the cognitive effects described in the literature in individuals with multimodal auras [23,24]. However, a strong argument can be made that a single wave of CSD is not the most parsimonious explanation for our results and cannot explain all our findings.

Firstly, the range of offset times seen in every participant indicates that there can be no unique trigger location for that individual within the parieto-occipital cortical space; instead, the initiation point must vary quite widely across episodes. Looking at individual within-modality aura descriptions, both in our participants and in the literature (e.g., [8]), it is evident that while in most migraineurs’ auras can affect either visual hemifield and either body side and do not always follow precisely the same pattern or the same starting point, nevertheless the nature of the experience is usually quite stereotyped. Migraineurs, including our participants, easily identify auras that are “not my typical aura”. In the present series, for example, P2′s somatosensory auras always affected only one arm and hand, P5′s almost exclusively the nose, and P1′s generally affected half the face, including the mouth along with the ipsilateral arm. Their visual auras were equally stereotyped. It seems unlikely that such similar activation patterns within sensory cortex would arise from CSD waves initiated at so many disparate locations.

Secondly, the range of IOTs discussed above for two auras within a hemisphere (3–55 min; E = 7 for purely unilateral auras) is very similar to the range seen for two auras exclusively restricted to contralateral hemispheres (2–45 min; E = 6) such as a visual aura restricted to left visual field, followed by numbness on the right side of the face. Leão and Morison [10] demonstrated quite convincingly that CSD can spread across the corpus callosum when triggered by a strong stimulus, and may indeed play a role in bilateral auras as we discuss below. However, the strength of CSD in the second hemisphere was usually weaker than the primary wave, so it would be very surprising for a wave of CSD initiated, for example, in the right visual cortex evoking a left visual field aura, to then die out in the right hemisphere but successfully cross the corpus callosum at some point, without evoking right visual field symptoms, and spread all the way to the face representation in the left somatosensory cortex. While not impossible, separate triggering in or near two hyper-excitable sensory areas does seem more probable.

#### 4.1.2. Does the Corpus Callosum Explain Bilateral Auras?

In his landmark papers describing experimentally induced cortical spreading depression, Leão [10,44] noted that when a strong stimulus was applied to the cortical surface in one hemisphere, a secondary wave of CSD was often triggered in a symmetrical location in the contralateral hemisphere. The progress of the second wave was always slightly delayed relative to the primary one but once initiated, the rate of spread within each hemisphere was similar [10]. Moreover, the transit time between hemispheres was much more rapid than the spread within each hemisphere. Physical separation of the two cerebral hemispheres above the corpus callosum with a glass sheet did not prevent interhemispheric transfer of CSD, ruling out direct diffusion of ions between hemispheres. However, severing the corpus callosum completely prevented the crossing. A final observation from Leão’s extensive work was that interhemispheric transfer was entirely related to the triggering event for a wave of CSD; in his studies, direct mechanical, chemical or electrical interference with the brain. With a strong trigger, both ipsilateral and (after a slight delay) contralateral waves were triggered from mirror-symmetric locations. If only an ipsilateral wave of CSD was triggered, it was never observed to cross to the other hemisphere at other points along its course. Can this sort of callosal transfer explain the bilateral aura symptoms in our dataset?

In human primary visual cortex, callosal fibers arise only along the V1/V2 border which represents the vertical meridian [45]. These connections have long been assumed to knit together our representation of the full visual field and to support functions involving midline, and especially foveal, vision. Two types of visual aura that involved crossing the vertical meridian were described by our participants. One individual reported zig-zag positive auras that she described and drew as moving around a clock face, circling from the lower to upper peripheral visual field. In some episodes, they bridged the vertical meridian and then moved down through the opposite hemifield (Figure 1a), strongly suggesting activity crossing the corpus callosum. However, it is important to note that this interhemispheric transfer did not occur at the initial triggering of the aura. Two individuals reported bilateral loss of peripheral vision in auras which followed (or co-occurred with) bilateral somatosensory auras. The mapping of the visual field loss was not precise enough to determine the timing of onset or spread in the two hemifields, but it generally began in the lower visual field and spread upward into both peripheral hemifields, leading to complete tunnel vision in some cases. Thus callosal spread could be involved.

A similar role of joining the midline has been proposed for callosal connections in the primary somatosensory cortex. Those areas (face, oral structures, trunk) where the surface is continuous across the midline would be expected to have strong callosal connections and numerous studies in humans and other primates supports this [46]. It was long assumed that the limbs would not have such connections, since they are not a continuous receptor surface, and in many (but far from all) functions, they operate independently. However, more recent work has clearly established that there is direct callosal connection between limb areas of SI as well [47,48], and that there are broad networks of functional interaction (mainly negative) between somatosensory areas in the two hemispheres operating between both homotopic and heterotopic areas [49,50]. It should also be noted that SII, a smaller region considered a higher level somatosensory area, that lies in the parietal operculum (i.e., below the face representation) is heavily endowed with callosal connections such that there is a complete ipsilateral body representation in this area, the input to which is supplied via the corpus callosum [51,52].

Nearly half (14/29) of the somatosensory auras reported in this study involved symptoms on both side of the body. In two episodes, the body regions affected were different—in each case, one facial region, and the opposite hand (Figure 2c). The remaining 12 bilateral auras consisted of sensory symptoms (generally tingling or numbness) affecting either the facial region or the limbs. Bilateral effects in one of these regions could be accompanied by bilateral, unilateral or no symptoms at all in the other region. When both upper limbs were affected (E = 9), the sensations were centred on identical areas (e.g., both hands), although symptoms on one side were frequently reported to be more intense or were drawn as somewhat more extensive (e.g., extending into the forearm; see Figure 2b). The apparent onset time of the less affected side was delayed in most cases. In two episodes the lower limbs were also affected, with the same pattern of symmetry. This pattern is what one would predict from Leão’s description of interhemispheric spread [10], and would be compatible with a triggering event in the hemisphere where symptoms appear first and are strongest.

It is more difficult to interpret bilateral symptoms in the face. Of the nine reports documenting bilateral facial auras, only two showed widespread symmetric involvement of both sides of the face at the outset of the aura. The remainder generally involved broad effects on one side of the face that gradually encroached across the midline to affect part of the other side, most often the upper or lower lip (Figure 2b). At present, the layout of the human facial region in SI remains poorly understood, especially the representation of the skin surface [53,54,55,56,57,58]. Penfield described ipsilateral/bilateral representation of some oral structures [19], and in non-human primates, there is substantial evidence for a bilateral map of oral structures in SI. Electrophysiological evidence from non-human primates [59,60,61] suggests that the ipsilateral representation may reflect direct input from the ventroposterior medial (VPM) nucleus of the thalamus rather than via the corpus callosum. The ipsilateral thalamic input is most clearly delineated for the lips and intraoral structures, with ipsilateral features mapping below contralateral areas. If the human SI organization turns out to be comparable to monkeys, CSD that spread further downward in the face region might be predicted to produce activation spreading to the ipsilateral lips, teeth and/or tongue, the pattern reported in some episodes in this study.

#### 4.1.3. What Can We Conclude about CSD and Sensory Auras?

The version of the CSD hypothesis least able to explain the present observations is that an aura is first initiated in one sensory cortical region (usually visual) and a wave of CSD spreads outward from that point in all directions, eliciting both the symptoms of a visual aura and at the same time spreading across non-sensory “silent” cortex until the somatosensory cortex is reached, at which point new sensory symptoms are elicited (e.g., tingling of the fingers). The timing between the start of the aura pairs was too brief in at least half the episodes for this to be a plausible explanation, and a multitude of different trigger points in the parietal cortex would be needed to explain all the temporal offsets seen, even within the same individual, in this small dataset. Moreover, a single CSD wave cannot easily explain a visual aura in one visual field followed by sensory symptoms limited to the opposite side of the body, nor the appearance of unilateral sensory symptoms in one region (e.g., face), and the simultaneous appearance of contralateral symptoms limited to a different region, for example, the hand or arm.

A similar conclusion has been reached from quite similar reasoning in a recent theoretical analysis and review of literature relating aura to spreading depression [62]. Bolay and colleagues question whether classical CSD, as described by Leão, underlies any of these aura phenomena, and suggest changes in thalamo-cortical connectivity as a critical factor in explaining aura. In our view, the phenomenology of fortification visual auras clearly requires a mechanism involving the spread of excitation and either inhibition or cortical depression, giving rise to a visual experience that reflects the intrinsic organization of receptive fields and local excitatory and inhibitory networks in V1 [63]. This might not be true CSD but the very slow rate of topographic spread is certainly suggestive of a diffusion process as the underlying cause. Although visual auras, especially fortifications, seem to have a stereotyped pattern, what appear to be very similar starting points near the fovea can actually be spread across a large cortical area because of the cortical magnification factor [33] (See Figure 2 of [8]), suggesting that where CSD actually pops up within a sensory “map” could be a somewhat random event depending on fluctuating local ionic imbalances. Similar considerations may apply in the somatosensory cortex—the areas with the largest representations are the most frequent site of aura initiation. The more restricted spread of somatosensory auras could reflect structural barriers to diffusion between subregions [64], which might also contribute to the prolonged auras reported here and elsewhere.

Clearly the transition from interictal status to prodromal symptoms to the onset of aura, headache and the other features of a migraine episode (nausea, photophobia etc) entails major changes in excitability of some brain regions. The simplest possibility, and in many respects the most appealing, would be that the excitability of sensory cortical areas is directly altered as a migraine episode unfolds (possibly due to changing thalamic modulation [62]), and the cytoarchitecture or local wiring of primary visual and somatosensory areas makes them especially prone to supporting CSD if it can be triggered. An alternative form of the independent initiation hypothesis would be that auras are initiated by high levels of activity in very specific regions of the cortex, such as area V3a that form part of a “migraine network”. The same considerations of timing would suggest that there must be multiple driver regions. Separate network nodes (e.g., SII discussed below) might play a crucial role here.

### 4.2. Functional Networks and Network Dysfunction

Another enormous advance in systems neuroscience over the past 25 years has been our growing understanding of functional networks in the brain, regions of the brain with strongly correlated activity during particular task-related functional states or at rest (e.g., [65]). This approach has been applied to migraine, most notably by Hougaard et al. [66] who were able to compare such network patterns in fMRI from patients scanned within 8 h of visual auras (and in some cases somatosensory and language-related auras) to the comparable scans of the same individual’s brain in a migraine-free period. Their principal findings were increased ictal connectivity between the left pons and the head and face regions of the left primary somatosensory cortex. When normalized for aura side, their data also revealed increased connectivity between visual area V5 (also known as MT; turquoise in Figure 4) and a region of the frontal cortex.

Lesion network mapping, introduced by Boes et al. ([67]; see [68] for an overview of this approach) takes advantage of the Human Connectome to ask how common symptoms in patients with disparate structural lesions may arise by uncovering key network nodes involved in disease states linking the lesion sites. While originally applied to explicit lesion data, the approach has been extended to more subtle regional abnormalities, such as reduced cortical thickness, to identify networks involved in neurological and behavioural abnormalities. Burke et al. [69] have recently applied this approach to migraine based on the growing body of reports of grey matter volume differences reviewed in a recent meta-analysis [70]. Despite the fact that most of the cases upon which this analysis was based involved migraine without aura, their most striking finding [69] was that all atrophy regions were connected (negatively) to the left extrastriate visual cortex, in particular areas V3 and V3a (see Figure 4), the area that Hadjikhani et al. [12] had previously identified as the earliest site of activation in the visual migraine auras captured by fMRI. The insula (bilaterally) was connected positively to most of the sites, and there was a strong negative connection with the hypothalamus, posterior thalamus and much of the visual cortex [69]. While the insula and hypothalamic sites may be more related to pain than to other migraine symptomology, as they did not distinguish between migraine and a control condition of chronic pain, the V3/V3a site appeared to be unique to migraine.

In a 2013 study, Hadjikhani et al. [71] drew attention to enhanced connectivity within the limbic/viscerosensory network in migraine with and without aura. Their focus was on linkage between the amygdala and the insula, highly involved in visceral and pain processing. However, they also reported almost equally enhanced connectivity between amygdala and the parietal operculum, site of secondary somatosensory cortex (SII, OP1 in [38], light green in Figure 4). Given our aura results, this area may merit further scrutiny. Similar to the primary somatosensory cortex (SI), this region contains somatotopic mappings of the body with some ipsilateral representation [72] and shows strong reaction to painful stimuli [73]. It has also been shown to be the somatosensory region most heavily connected through the corpus callosum [74]. Similar to area V3a (mauve in Figure 4), the surface area of SII is about 2 cm^2^ [52], much smaller than that of SI, and probably is not itself the main source of somatosensory aura phenomenology; nevertheless, similar to V3a, it could play a key role in initiation CSD. As our data suggests the possible role of callosal transfer of CSD-like activity to mirror image representations, this is an important point for further investigation.

The field of functional networks is developing rapidly; as with any new findings, replication will be essential to confirm which of the broad range of fMRI-defined “connections” [13] prove to be both replicable and meaningful. This is especially true of laterality effects, the interpretation of which is generally not obvious. However, improved descriptions of auras in the same individuals in whom imaging is performed would be a valuable advance.

### 4.3. Aura Complexity—Comparison to Recent Studies

The present case reports show a picture very similar to that presented by the large series of retrospective and prospective cases of migraine with aura reported by Viana and colleagues [16,17,31] and also the cases discussed by Petrusic et al. [25,75] in developing their Complex Aura scale. Visual auras of our participants showed the same types of positive (flashes of light, shards of light, zig-zag lines) and negative (missing areas, dark/grey, blurred areas) phenomena described in these large studies. Aura durations varied both within and across individuals (Table 3); the range of median durations was 15–65 min. Prolonged visual auras were rare—only 2 episodes (6.8%) exceeded the 60 min specified in the current ICHD-3 criteria [32]. This low percentage is consistent with what has been reported by Viana both in a literature review [31], and in a prospective diary study of individuals with auras in multiple modalities [17].

Viana et al. [17] reported a higher proportion (21%) of prolonged somatosensory auras than visual auras in individuals experiencing auras in multiple modalities, and our findings agree with this observation. Three of our six participants experienced prolonged somatosensory auras (>60 min) on multiple occasions (16 episodes in total; see Table 4 and Supplementary Table S1 for details). It may prove valuable in the future to pay careful attention to differences between positive (tingling, pins and needles) and negative (numbness) symptoms in very long auras, as the latter may indicate a very long suppression of cortical activity which could occur with CSD, whereas positive features would be expected early (positive wavefront of CSD [11]), and possibly also much later as recovery occurs and spontaneous activity returns to normal levels.

While we were unable to document the precise timing of headache in all cases, our data generally accord with the observations of Viana et al. [16] in that we have seen instances of headache preceding, co-occuring with, and following the auras. Headache was documented in all 29 episodes that make up this report; however, three of these individuals reported that they occasionally experienced aura symptoms without a full headache.

Petrusic and colleagues [25,75] have recently argued for a classification of auras that takes complexity (multiple auras and higher level effects) into account, and have developed the Migraine Aura Complexity Scale (MACS) to assess this. Because we wanted our participants to focus on the timing and phenomenology of the two sensory auras, we did not ask them to record cognitive disturbances in this study, although four of them had reported these at interview; however, in some episodes detailed comments were included about such symptoms. Based on this information, two of our participants would meet the 4.5 point cut-off for complex auras on some episodes. While sensory auras, as explored here, offer some promise as a tool to evaluate the CSD hypothesis, higher level functions are much more difficult to track in time. As they generally reflect disrupted function, somewhat analogous to scotoma or numbness, they are only detected if the individual tries to perform the function. While it is hard to miss a visual fortification aura, you will only be aware of a problem with calculation if you are doing math, or with speech production if you try to speak. Developing tasks that explicitly test for these deficits will be critical to evaluating the time course of disturbance of such complex functions. However, the MACS [25,75] will be an excellent tool for identifying individuals for whom such detailed assessments could be developed.

### 4.4. Limitations

Clearly the findings reported here are limited by constraints imposed by the technology available at the time. Twenty-five years ago, smart phones did not exist, email was not widely available, the internet was in its infancy and relatively few people had home desktop computers. Thus, pen and paper data collection and mailed-in responses were the best alternative for data recording outside the laboratory.

The greatest shortcoming of the study is clearly the small number of participants. Our initial interest was in visual auras and we only gradually came to appreciate that some of the migraineurs we were seeing also had somatosensory auras. Our visual mapping procedure, designed to capture the precise location of aura components in the visual field had proved very effective for mapping visual auras when that was the only requirement, but proved to be difficult for some of the V-SS participants to accomplish, especially if their two auras overlapped in time. Moreover, practical considerations of map size meant that our maps did not capture events in the far visual periphery, which turned out to be quite important in this group. The somatosensory maps proved much easier to use. Their greatest limitation was that the hand region on the maps was very small despite the fact that the hand occupies a very large area of the somatosensory cortex [19] with each digit and thumb having a large separate representation [37,48]. While at least one participant tried to make these fine distinctions, some simply indicated that a hand was affected, so this may have obscured valuable evidence of the pattern of local aura spread.

Our emphasis on recording data only as the episodes were ongoing, and mailing the results back immediately made it likely that this instruction was followed but, of course, one can never be completely certain. The maps and reports were dated and usually mailed in promptly, which prevented participants from comparing their own maps and comments across episodes. Furthermore, our laboratory was an independent group, and while participants were typically referred by a neurologist, it was clear to them that we were not part of their care team. Therefore, unintended demand characteristics which might influence reports to their own physicians were less likely. While this may have led to their reporting only a subset of all their migraine episodes, we believe it also reduced the likelihood that data was recorded retrospectively, as might happen if an individual fell behind on a headache diary. All our participants had a long migraine history, had sought help, often at several levels of the medical system, and seemed eager to contribute to research that might eventually lead to alleviation of their suffering. The fact that they continued to contribute data to the mapping projects for up to four years attests to this.

### 4.5. Twenty-Five Years Later—Going Forward

If we were to undertake this study today, we would do many things differently. Modern technology offers so many possibilities. However, to date they have not been applied as they might have been to these questions, so we hope that our findings will stimulate others to pursue this problem further using a similar approach. The difficulties of capturing a migraine aura in an fMRI scanner are no less difficult today than they were at the time of Hadjikhani’s landmark report [12]—the lack of predictability of episodes, and ethical questions surrounding triggering auras, even if a reliable trigger were known [76]. However, at the very least, fMRI imaging of stimulation of the body part exhibiting symptoms during a typical aura episode along with visual stimulation of the visual hemifield most often involved, using stimuli similar to patterns described during auras [77] could provide a basis for evaluating distances of spread when episodes do occur.

The range of possibilities for tracking migraine auras as they progress is now enormous. The smart phone or tablet, with appropriately designed applications could overcome many of the difficulties we encountered, and indeed one recent report has used electronic diaries [22]. In addition to checklists of symptoms, one could also envision the use of electronic maps of both visual field and body regions, time stamped and recorded as data when the observer was satisfied with their responses, and then recycled at 5–10 min intervals. Smart phone audio recording applications open up the possibility of recording verbal symptom descriptions as they progress for later transcription by researchers; this approach could also be valuable in the study of other hallucinations such as those experienced in Charles Bonnet syndrome [4]. The point is that, with the development of suitable software, the observer’s symptoms could easily be recorded electronically and then transmitted immediately to the researcher, ruling out any retrospective enhancement of the record.

## 5. Conclusions

These six cases are presented here in the spirit of contributing to the growing picture of the complexity of migraine aura. Their reports are not meant to provide a fully representative overview of even their own migraine condition, as a prospective diary study might have done. What they do reveal is detailed timing, laterality and qualitative information about a selection of episodes that occurred at times and in places in which a careful documenting of the auras in progress was possible. As such, they complement the findings of retrospective and prospective studies of much larger clinical populations and highlight issues that remain unresolved in the literature twenty-five years later. We hope they will generate further research into migraine auras, which still hold the promise of valuable insights into the underlying mechanisms of migraine.

## Figures and Tables

**Table 1:** Participant information.

Participant:	P1	P2	P3	P4	P5	P6
Gender (M/F)	F	F	M	F	F	F
Age at study onset (years)	44	24	33	43	63	46
Migraine History (years)	4	10	4	25	13	18
Handedness	Right	Not recorded	Right	Right	Right	Right
Family history of migraine	None known	Yes maternal	Yes paternal	Not recorded	Not recorded	Yes
Language for test materials	French	English	English	French	French	French

**Table 2:** Initiation offset times (IOT) in minutes and laterality information about auras and headache.

a. Aura IOT (min)—Positive Values Indicate Visual Aura Began First						
Participant:	P1	P2	P3	P4	P5	P6
**Episode #**						
1	3	5	−25	20	20	−10
2	23	30		2	45	0
3	5			55	17	−40
4	10			75	10	0
5				50	25	−165
6				27	5	0
7					30	−30
8						−15
9						−60
**b. Symptom laterality (Visual—Somatosensory—Headache)**						
**Participant:**	**P1**	**P2**	**P3**	**P4**	**P5**	**P6**
**Episode #**						
1	L ^1^-L-B	L-L-L	B-B-L	L-L-R	L-R-B	R-R-R
2	B-L-B	L-L-L		R-L-L	L-B-R	B-B-B
3	R-L-L			R-R-R	L-B-B	R-B-R
4	L-B-B			B-L-R	L-B-R	R-B-B
5				L-L-L	L-R-R	B-B-R
6				R-L-B	R-B-B	B-B-B
7					L-B-R	B-B-B
8						R-B-R
9						R-B-R

^1^ L = left, R = right, B = bilateral.

**Table 3:** Visual aura characteristics.

Participant:	P1	P2	P3	P4	P5	P6
Median duration	39 min	65 min	>20 min	15 min	25 min	45 min
(range)	(13–45; N = 4)	(25–105; N = 2)	N = 1	(5–35; N = 6)	(17–35; N = 7)	(15–210; N = 7)
Positive Features	Zig-zag around clock face	Yellow spots, shards	Lines/ diamonds	Scintillating, pulsing (rare)	Fortification	Bright flashing areas (rare)
Negative Features	None reported	None reported	Facet-like tunnel vision	Dark moving cloud	Moving scotoma	Peripheral Grey/dark/ blur

**Table 4:** Somatosensory Aura Characteristics.

Participant:	P1	P2	P3	P4	P5	P6
Median duration (min)	78.5 min	52.5 min	16 min	21.5 min	200 min	105 min
(range)	(42–105; N = 4)	(45–60; N = 2)	(15–17; N = 2)	(15–60; N = 6)	(5–420; N = 6)	(50–660)
Principal sites of SS aura symptoms	Arm, face, mouth	Arm, hand	Face (lips), hand	Face (lips), hand	Nose, limbs	Fingers, hand, face, mouth

## Data Availability

Due to privacy concerns individual aura reports and maps will not be made generally available as they often contained identifying information or personal information not relevant to the focus of this study. The author will provide anonymized copies of material where possible upon person request.

## Supplementary Materials

The following are available online at https://www.mdpi.com/article/10.3390/vision5040052/s1, Figure S1. Visual and somatosensory map forms. Figure S2. Visual aura data form. Figure S3. V-SS aura data recording form, Table S1. Episodes evaluated by ICHD-3 criteria.
