# Supplementary material for: Aura Mapping: Where Vision and Somatosensation Meet
Source: Vision (Basel). 2021 Oct 30;5(4):52. doi: 10.3390/vision5040052 (PMC8628888; doi:10.3390/vision5040052)
Supplement: Supplementary file 1 [file vision-05-00052-s001.zip › Supplementary Figure S1.pdf]

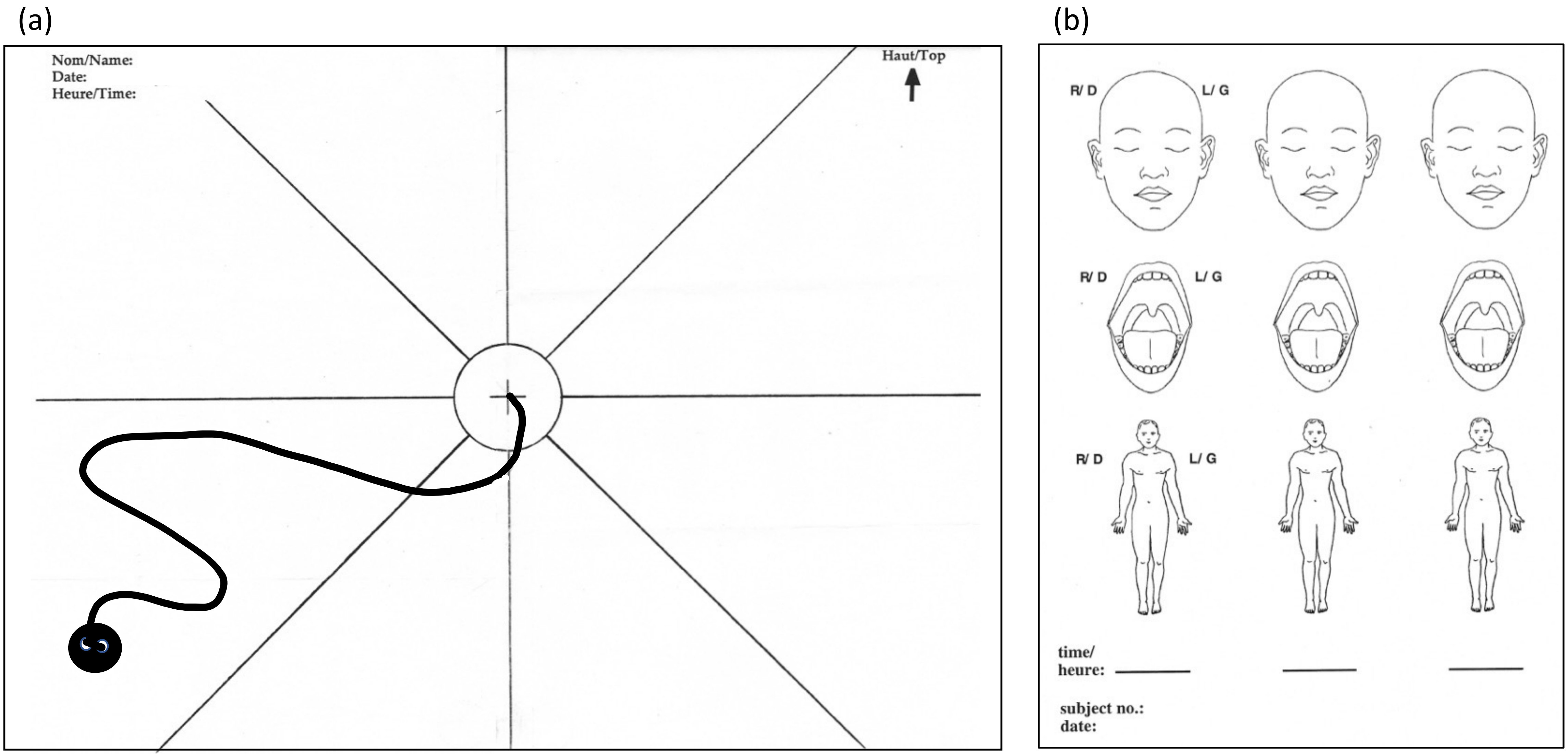

Figure S1: (a) Form for visual aura map. The string extending from the fixation + was 40 cm in length. The participant was instructed to use the string to position themselves at the correct viewing distance by pulling the button to the bridge of their nose. (b) A set of three somatosensory maps used to map the spread of somatosensory aura symptoms.
