# Supplementary material for: Aura Mapping: Where Vision and Somatosensation Meet
Source: Vision (Basel). 2021 Oct 30;5(4):52. doi: 10.3390/vision5040052 (PMC8628888; doi:10.3390/vision5040052)
Supplement: Supplementary file 1 [file vision-05-00052-s001.zip › Supplementary Figure S2.pdf]

Supplementary Figure S2: English version of the form used for the Visual Aura Mapping Study. (French version available from the author on request: franw@yorku.ca)

MIGRAINE RESEARCH PROJECT - McGILL UNIVERSITY

Migraine Aura Observation Form

Date:______________________________ **Subject #** __________

******* FILL OUT THIS FORM ONLY DURING A MIGRAINE EPISODE *******

1. On which **side** do you see the aura? left ____ right____ both ____
2. **Time** at **beginning** of aura: ___________ (to the nearest minute)
   Time at **end** of aura: ____________ (to the nearest minute)
3. **Observe your aura carefully**, preferably in a dim room, and **describe what you see:**
   spots ____ zig zag lines ____ other lines ____
   other (describe)____
   Please try to **draw an example** of what you see on the back of this form

   the spots / zig zag / lines / other are: bright ____ dark ____ both ____
   (pay particular attention to whether there are dark elements)

   do you see: blind area(s), missing parts yes___ no___
   blurred area(s) yes___ no___

   the aura is: flashing / flickering / rolling yes___ no___
   (if yes, circle which description applies best)
   moving or drifting across the visual field yes___ no___

   do you see any colour? (excluding black and white) yes___ no___
   if yes, which ones? ______________________________
   If possible **draw or describe** the form/shape of coloured areas on the back of this form.
4. Attach the **map** to folder or wall. Use the string and button to **position your eyes at the correct distance** (this is very important).
   While fixating the central cross:
   - Mark the position where you first see the aura.
   - At 5 min intervals, mark the position of the aura and write the time beside the mark (always fixate on the cross while marking position).
5. Please fill out **during your headache** (if any):

   Do you have a headache? yes___ no___
   if yes: on which side? left ____ right ____ both ____
   when did it start? ___________
   how long did it last? ______________

- Did you experience any other unusual sensations (sounds, smells, tastes, numbness, tingling) during your migraine episode? yes___ no___. If yes, please describe them on the back of this form.
- If there was anything striking or unusual about your visual aura please make a note of this on the back.
- Please indicate on the back of the form anything that you think might have triggered this episode.

PLEASE MAIL THIS FORM BACK AS SOON AS POSSIBLE

Back of form:

Medications:

Did you take any medication during this episode? yes ____ no____

Which ones:____

When:______________________________
