# Supplementary material for: Aura Mapping: Where Vision and Somatosensation Meet
Source: Vision (Basel). 2021 Oct 30;5(4):52. doi: 10.3390/vision5040052 (PMC8628888; doi:10.3390/vision5040052)
Supplement: Supplementary file 1 [file vision-05-00052-s001.zip › Supplementary Figure S3.pdf]

Supplementary Figure S3: English version of the form used for the Visual – Somatosensory Mapping Study. (French version available from the author on request: franw@yorku.ca).
Visual Aura Question 4 and Headache Question 3 were added to the questionnaire as the study was in progress when it became clear that some participants could not map the visual aura in the same episode that they were mapping a somatosensory aura.

MIGRAINE RESEARCH PROJECT - McGILL UNIVERSITY

Migraine Aura Observation Form

Date:______________________________ Subject #: __________

******* FILL THIS FORM OUT ONLY DURING A MIGRAINE *******

VISUAL AURA

1. On which side do you see the **visual** aura?
   left ______ right______ both ______

2. Time at beginning of **visual** aura: ___________
   Time at end of **visual** aura: ____________

3. Attach map to folder or wall. Use the string to position your eyes at correct distance (this is very important).

   While fixating central cross:
   - Mark the position where you first see the aura.
   - At 5 min intervals, mark position of aura and write time beside mark (always fixate cross while marking position).

4. Is this visual aura like those you have had previously? yes____ no____
   Describe your aura very briefly on the back of this form (dots, zig-zags, flicker/movement, blurred areas etc) noting any unusual features.

SENSORY AURA

1. On which side of your body do you feel the **sensory** aura?
   left ______ right______ both ______

2. Time at beginning of **sensory** aura: ___________
   Time at end of **sensory** aura: ____________

3. On the sensory aura map, indicate where you feel numbness, tingling or other bodily sensations.
   - Note on the map where you initially feel these sensations.
   - At 5 min intervals, note the location of the aura and write the time below each observation (use more than one form if necessary).

HEADACHE (please fill in while experiencing the headache)

1. Do you have a headache? yes____ no____

2. If so, on which side? left ______ right ______ both ______

3. When did it start? __________ How long did it last? ________

If there was anything striking or unusual about your migraine episode please make a note of this on the back of this form.

PLEASE MAIL THIS FORM BACK AS SOON AS POSSIBLE
