# Supplementary material for: Aura Mapping: Where Vision and Somatosensation Meet
Source: Vision (Basel). 2021 Oct 30;5(4):52. doi: 10.3390/vision5040052 (PMC8628888; doi:10.3390/vision5040052)
Supplement: Supplementary file 1 [file vision-05-00052-s001.zip › Supplementary Table S1_readonly.pdf]

Supplementary Table S1: Episodes evaluated by ICHD-3 criteria. For each recorded episode, each ICHD-3 aura characteristic is evaluated as YES (clearly met), NO (clearly fails), or INC (inconclusive - insufficient information provided to evaluate). Durations of prolonged auras (>60 min) in red. Overall rating for episode (top line) based on criterion of at least three characteristics met.

**Participant 1**	**Episode 1**	**Episode 2**	**Episode 3**	**Episode 4**	**Episode 5**	**Episode 6**
At least three of the following six characteristics:	**YES**	**YES**	**YES**	**YES**		
at least one aura symptom spreads gradually over ≥5 minutes	YES SS maps	YES SS maps	YES from SS maps	Yes from SS maps		
two or more aura symptoms occur in succession	YES V,SS Offset 3 min	YES V,SS. Offset 23 min	YES V,SS Offset 5 min	YES V,SS Offset 10 min		
each individual aura symptom lasts 5-60 minutes	YES (V=13; SS=42)	NO (V=34; SS=92)	NO (V=44; SS=105)	NO (V=45; SS=65)		
at least one aura symptom is unilateral	YES both (L)	YES SS (L)	YES V (R), SS (L)	YES. V (L)		
at least one aura symptom is positive	INC	INC	INC	INC		
the aura is accompanied, or followed within 60 minutes, by headache	INC HA(B) no time; Meds within 60 min	INC HA(B) (no onset time)	INC HA(L) (no onset time) Meds during aura	INC HA(B) (no time) Meds within 60 min		
**Participant 2**						
At least three of the following six characteristics:	**YES**	**YES**				
at least one aura symptom spreads gradually over ≥5 minutes	YES (SS maps)	YES V and SS				
two or more aura symptoms occur in succession	YES. V, SS Offset 5 min	YES V, SS Offset 30 min				
each individual aura symptom lasts 5-60 minutes	YES (V=25; SS=45)	NO (V=105; SS=60)				
at least one aura symptom is unilateral	YES V (L), SS (L)	YES V (L), SS (L)				
at least one aura symptom is positive	YES V, SS	YES V, SS				
the aura is accompanied, or followed within 60 minutes, by headache	INC – HA(L) (no onset time)	INC- HA(L) (no onset time)				
**Participant 3**						
At least three of the following six characteristics:	**PROBABLE**					
at least one aura symptom spreads gradually over ≥5 minutes	YES (SS maps)					
two or more aura symptoms occur in succession	YES SS, V Offset 25 min					
each individual aura symptom lasts 5-60 minutes	INC SS=15; V unknown (>20) sleep					
at least one aura symptom is unilateral	NO both bilateral					
at least one aura symptom is positive	INC tunnel vision, numbness					
the aura is accompanied, or followed within 60 minutes, by headache	INC awoke with HA(L) within 2 hr					
**Participant 4**						
At least three of the following six characteristics:	**YES**	**YES**	**YES**	**YES**	**YES**	**YES**
at least one aura symptom spreads gradually over ≥5 minutes	YES (SS maps)	INC – can't evaluate spread from map	YES (SS maps)	INC SS noted absence of spread; no V info	INC SS noted absence of spread	INC no info provided
two or more aura symptoms occur in succession	YES V,SS Offset 20 min	YES V,SS Offset 2 min	YES V,SS Offset 55 min	YES V,SS Offset 75 min	YES V,SS Offset 50 min	YES V,SS Offset 27 min
each individual aura symptom lasts 5-60 minutes	YES (V=20; SS=60)	YES (V=15; SS=23)	YES (V=15; SS=20)	YES (V=5; SS=20)	YES (V=35; SS=15)	YES (V=15; SS=33)
at least one aura symptom is unilateral	YES both L	YES V(R), SS(L)	YES both R	YES SS(L)	YES both L	YES V(R), SS(L)
at least one aura symptom is positive	INC	INC	INC	YES V	YES V	INC
the aura is accompanied, or followed within 60 minutes, by headache	INC HA(L) (no time); Meds during aura	YES HA(L) during aura	YES HA(R) during aura	INC HA(R) (no time); Meds during aura	INC HA(L) (no time); Meds during aura	YES HA(Bilat)
**Participant 5**						Sept 18, 1996 #10
At least three of the following six characteristics:	**YES**	**YES**	**YES**	**YES**	**YES**	**YES**
at least one aura symptom spreads gradually over ≥5 minutes	YES V	YES V	YES V	YES V	YES, V	YES V
two or more aura symptoms occur in succession	YES V, SS Offset 20 min	YES V,SS. Offset 45 min	YES V,SS Offset 17 min	V,SS Offset 10 min	YES V,SS Offset 25 min	YES V,S Offset 5 min
each individual aura symptom lasts 5-60 minutes	NO (V=25; SS = 240)	INC (V=25; SS end time not recorded)	YES (V=17; SS=5)	NO (V=35; SS= 420)	NO (V=30; SS = 200)	NO (V=30; SS=80)
at least one aura symptom is unilateral	YES V(L), SS(R)	YES V(L)	YES V(L) (SS bilateral face)	YES V(L), (SS limbs Bilat face R)	YES (L), SS(R)	YES V(R), SS limbs bilat
at least one aura symptom is positive	YES V, SS	YES V,SS	YES V,SS	YES V,SS	YES V,SS	YES V
the aura is accompanied, or followed within 60 minutes, by headache	YES HA(B)	YES HA(R)	YES HA(B)	YES HA(R)	YES HA(R)	YES HA(B)
**Participant 6**						
At least three of the following six characteristics:	**YES**	**YES**	**YES**	**YES**	**NO**	**YES**
at least one aura symptom spreads gradually over ≥5 minutes	YES V, SS maps	YES V, SS shift from bilateral to left	YES SS maps	YES SS maps	INC do not have map for first 2 hr	YES V, SS
two or more aura symptoms occur in succession	YES (SS, V) Offset 10 min	NO Simultaneous onset; SS lasted longer	YES (SS, V) Offset 40 min	NO Simultaneous onset; SS lasted longer	YES (SS, V). Offset 285 min	NO Simultaneous onset;
each individual aura symptom lasts 5-60 minutes	NO (V=40; SS=100)	YES (V=40; SS=50)	NO (V=50; SS=120)	NO (V=45; SS=95)	NO (V=15; SS = 11 hours)	NO (V>35; SS=105)
at least one aura symptom is unilateral	YES V(R), SS(R)	NO both start bilateral; then stronger left	YES V(R)	NO both bilateral	NO both bilateral	NO both bilateral
at least one aura symptom is positive	YES SS	YES SS	NO	YES V	NO numbness & tunnel vision	YES V
the aura is accompanied, or followed within 60 minutes, by headache	YES HA(R) started with aura	INC HA(B) onset time not noted	YES HA(R) during visual aura	YES HA(B) during auras	YES HA(R) 3 hr before aura; lasted all day	YES HA(B) during auras

Supplementary Table S1 (Cont'd)

	Episode 7	Episode 8	Episode 9			
**Participant 5**						
At least three of the following six characteristics:	**YES**					
at least one aura symptom spreads gradually over ≥5 minutes	YES V					
two or more aura symptoms occur in succession	YES V,S Offset 30 min					
each individual aura symptom lasts 5-60 minutes	NO (V=20; SS=240)					
at least one aura symptom is unilateral	YES V(L), (SS B face and limbs)					
at least one aura symptom is positive	YES V, SS					
the aura is accompanied, or followed within 60 minutes, by headache	YES H(R)					
**Participant 6**						
At least three of the following six characteristics:	**YES**	**YES**	**YES**			
at least one aura symptom spreads gradually over ≥5 minutes	YES SS maps	YES V, SS maps	YES V,SS maps			
two or more aura symptoms occur in succession	YES SS, V Offset 30 min	YES SS, V Offset 15 min	YES SS,V Offset 60 min			
each individual aura symptom lasts 5-60 minutes	NO (V=180 min; SS = 240 min)	NO (V=50; SS=85)	NO (V=>40 (sleep); SS-105)			
at least one aura symptom is unilateral	NO both bilateral from maps	NO both bilateral (face R, hand L, V R>L)	YES V(R)			
at least one aura symptom is positive	YES SS	YES V, SS	NO numbness and blurred grey areas of vision			
the aura is accompanied, or followed within 60 minutes, by headache	YES HA(R) during auras	YES HA(B/R) 10 min after 2nd aura	YES HA(R) preceded and during SS aura			
	B-B-R	B-B-B	R-B-R			
